# Long-term experience with idursulfase beta (Hunterase) in two adolescent patients with MPS II: A case series

**DOI:** 10.1016/j.ymgmr.2023.100991

**Published:** 2023-07-12

**Authors:** Mei-Yan Chan, Andrew Jack Nelson, Lock-Hock Ngu

**Affiliations:** Department of Genetics, Hospital Kuala Lumpur, Malaysia

**Keywords:** Mucopolysaccharidosis, Asian, Enzyme replacement therapy, Idursulfase beta

## Abstract

Mucopolysaccharidosis (MPS) type II (Hunter syndrome) is a rare X-linked, recessive, lysosomal storage disorder caused by the deficit of the enzyme iduronate 2-sulfatase (IDS), resulting in accumulation of glycosaminoglycans (GAGs) impairing cellular function in multiple organ systems. Idursulfase (Elaprase, Takeda Pharmaceuticals) and idursulfase beta (Hunterase, GC Biopharma Corp.) are the two currently available enzyme replacement therapies (ERT) for MPS II in Malaysia. ERT in patients with MPS II is associated with improvements in somatic symptoms, pulmonary function, endurance, joint mobility, and quality of life. Though mostly well tolerated, infusion-associated reactions (IARs), such as allergic (IgE-mediated) or nonallergic (non- immunologic) reactions can develop during ERT. In certain cases, when patients develop recurrent IARs despite reduced infusion rate and premedication, either interruption or cessation of ERT might be necessary. However, interruption of ERT is associated with worsening of clinical symptoms such as recurrent respiratory infections, difficulty in standing and walking, and increased joint stiffness, emphasizing the need for continuation of ERT. Here we report successful long-term experience with the use of idursulfase beta in two adolescent Malaysian patients with MPS II, who experienced recurrent infusion-associated reactions warranting discontinuation of ERT with idursulfase.

## 1 Introduction

Mucopolysaccharidosis (MPS) type II, also known as Hunter syndrome, is a rare X-linked, recessive, lysosomal storage disorder caused by the deficit of the enzyme iduronate 2-sulfatase (IDS). MPS II results from alterations in the *IDS* gene (HGNC ID:5389; ENSG00000010404), which is responsible for providing instructions to produce iduronate 2-sulfatase (D'Avanzo et al. Int. J. Mol. Sci. 2020;21:1258). [1] Commonly reported variations in *IDS* gene include missense/nonsense mutations followed by deletions, splice site mutations and insertions (Khan et al. Mol Genet Metab. 2017;121(3):227–240). [2] The birth prevalence of MPS II is 1.3 per 100,000 Caucasian male live births (Wrath et al. Eur. J. Pediatr. 2008; 167:267–277). [3] In Asian countries, the birth prevalence of MPS II ranges from 0.74 per 100,000 live births in South Korea and 0.84 per 100,000 live births in Japan to 1.07 per 100,000 live births in Taiwan. In countries like China, Japan, South Korea, and Taiwan, MPS II contributes to half of all MPS cases reported (Khan et al. 2017). [2] MPS II being an X-linked disorder mainly affects males. However, rare cases of heterozygous females demonstrating findings of MPS II have been reported (Khan et al. 2017). [2]

As *IDS* is a gene with ubiquitous expression, MPS II manifests as a multisystemic disorder. Since the lysosomal enzyme IDS is crucial for hydrolysis of 2-sulfate groups of the glycosaminoglycans (GAGs), dermatan sulfate (DS) and heparan sulfate (HS), deficit of the enzyme IDS, results in accumulation of these GAGs impairing cellular function in multiple organ systems (D'Avanzo et al. 2020). [1] Accumulation of GAGs results in typical clinical characteristics of MPS II such as coarse facial features, short stature, and joint stiffness, inguinal and umbilical hernia, thickening of heart valves, repeated respiratory tract infections, organomegaly and hearing impairment (Raiman et al. 2011. Canadian Consensus Statement). [4] MPS II presents across a wide spectrum of clinical severity ranging from attenuated to severe forms, mainly depending on the presence or absence of progressive neurological involvement and behavioral problems (Conner et al. Mol Genet and Metab Rep. 2019;21:100499). [5] Patients with attenuated disease comprise one-third of the cases, characterized by gradual onset and absence of cognitive involvement, with life expectancy up to fourth to sixth decade (Lampe et al. J Inherit Metab Dis. 2014;37:823–829). [6]

About two-thirds of patients have severe disease presenting at 2–4 years of age with chronic ear infections, facial dysmorphism, enlarged tonsils and tongue and enlarged liver and spleen. There is progressive cognitive impairment with behavioral disturbances such as hyperactivity, being easily frustrated, and impulsiveness along with severe speech and language delay (Conner et al. 2019). [5] Untreated patients with severe disease have a life expectancy only up to second or third decade (Lampe et al. 2014). [6] In addition to the significant clinical burden, severe MPS II imposes an enormous economic and social strain on families due to increased healthcare costs, caregiver time, need for social service assistance, special educational needs for the child, and adverse impact on parental work and productivity (Conner et al. 2019). [5]

The diagnosis of MPS II is established by demonstration of reduced or absent IDS enzyme activity in peripheral blood leukocytes, cultured skin fibroblasts, plasma or dried blood spots. This is followed by confirmatory genetic testing by identifying a hemizygous pathogenic variant in the *IDS* gene. (Raiman et al. 2011). [4] Quantitative analysis of urine GAGs shows increased excretion of dermatan sulfate and heparan sulfate in patients with MPS II, however this profile can also be seen in patients with MPS I.

Until recently, palliative care and symptomatic surgical interventions were the only available choices for management of patients with MPS II. Current treatment options include enzyme replacement therapy (ERT) and hematopoietic stem cell transplantation (HSCT). Though HSCT has demonstrated superior improvement in quality of life, limited evidence on its efficacy on neuropsychiatric outcomes and increased susceptibility to infection, especially in immunocompromised patients, limit the use of HSCT.

ERT with idursulfase (Elaprase, Takeda Pharmaceuticals) and idursulfase beta (Hunterase, GC Biopharma Corp.) are the two currently available enzyme replacement therapies for MPS II in Malaysia, administered as a weekly intravenous (IV) infusion at 0.5 mg/kg body weight over 1 to 3 h (Ngu et al., Mol Genet and Metab Rep. 2017;12:28–32). [7] ERT results in significant somatic improvement such as decline in the frequency of respiratory infections, reduction in the coarseness of facial features, and improvement in joint motion, especially when started earlier in the course of disease. However, ERT is of limited use in patients with cognitive decline because recombinant enzymes do not cross the blood-brain barrier, and consequently do not have any influence on central nervous system symptoms (Stapleton et al. Expert Opin Orphan Drugs. 2017;5(4):295–307). [8]

Both idursulfase and idursulfase beta are derived from the human *IDS* gene (NM_000202) and have the exact same amino acid sequence. However, there are certain differences between these two enzymes. Idursulfase is from human fibrosarcoma cell line in serum-containing medium whereas idursulfase beta is produced in Chinese hamster ovary cell line in a serum-free medium. (Kim et al. J. Hum. Genet. 2017;62:167–174). [9] Idursulfase beta demonstrates significantly higher enzymatic activity, faster cellular uptake (*in vitro* study using patient's fibroblast) and higher intracellular GAG degradation and lower anti-drug antibody formation (*in vivo* study using mice) compared to idursulfase (Kim et al. 2017). [9] The differences in manufacturing process and production in different cell lines result in differences in glycosylation process, which is critical for ERT of lysosomal enzymes. Idursulfase beta demonstrates significantly higher *in vitro* GAG degrading activity at lower doses compared to idursulfase (Kim et al. 2017). [9] The higher enzymatic activity of idursulfase beta is evident by the higher formylglycine (FGly) content of idursulfase beta (79.40%) compared to idursulfase (68.12%) (Kim et al. 2017). [9]. Though both idursulfase and idursulfase beta result in reduction in urine GAGs, idursulfase beta was associated with significantly greater reduction in kidney and lung GAGs (Kim et al. 2017). [9]

While ERT is mostly well tolerated, infusion-associated reactions (IARs), such as allergic (IgE-mediated) or nonallergic (non-immunologic) reactions have been documented. IgE mediated reactions occur immediately after the infusion and are characterized by cutaneous (flushing, rash), respiratory (swelling of tongue/uvula, dyspnea, wheeze-bronchospasm, stridor), gastrointestinal (abdominal cramps, vomiting), and systemic manifestations (reduced blood pressure, syncope). Therefore, antihistamine premedication is used to prevent these IgE mediated reactions. Non-IgE-mediated (anaphylactoid) reactions presenting as fever, chills, respiratory distress, tachycardia, nausea, and abdominal pain usually occur in the first hour after the infusion rate is increased. Reducing the rate of infusion is beneficial in such situations. Angioedema and acute airway obstruction, though rare, can be potentially serious complications of ERT, and require immediate cessation of the infusion and urgent resuscitation (Ngu et al. 2017). [7]

One of the challenges of ERT is the need for long-term chronic infusions, which may be responsible for the development of anti-drug antibodies. 50% of patients treated with idursulfase are known to develop anti-idursulfase antibodies within the first year of treatment. (Julien et al. Front Immunol. 2020; 11:1000). [10] Furthermore, 21% to 35% of these patients develop neutralizing IgG anti-drug antibodies to idursulfase, which reduce the efficacy of ERT and result in poorer clinical outcomes (Julien et al. Front Immunol. 2020; 11:1000). [10] The presence of anti-idursulfase IgG antibodies is linked to the development of hypersensitivity reactions, especially in younger patients with severe forms of mutations in the *IDS* gene (Gragnaniello et al. Mol Genet Metab Rep. 2022;31:100878). [11] *In vivo* study demonstrates higher concentration of anti-drug IgG antibodies in idursulfase treated group compared to idursulfase beta, (Kim et al. 2017) [9] which might be responsible for hypersensitivity reactions seen in patients on idursulfase ERT. However, no linear correlation between the level of anti-drug antibodies and risk of hypersensitivity reactions has been documented.

Furthermore, differences in cell lines, cell cultures, and purification, manufacturing, and fermentation processes may contribute to differences in immunogenicity of idursulfase and idursulfase beta. The manufacturing process of idursulfase involves the use of bovine serum, while idursulfase beta is manufactured using serum free chemical media, which results in better reproducibility and consequently reduced variability in immunogenicity across batches. Moreover, lower proportion of high-mannose type glycosylation in idursulfase beta, may be responsible for better immunogenicity profile of idursulfase beta. Additionally, higher cellular uptake of idursulfase beta can reduce the time it remains in the blood and thus reduce the subsequent immune response (Ngu et al. 2017). [7]

In some patients with recurrent IARs despite the reduced infusion rate and premedication, either interruption or cessation of ERT might be necessary. However, once ERT is started it is crucial to continue the scheduled administration frequency to maintain the clinical benefits. Discontinuation of ERT is not only associated with loss of clinical benefits but also worsening of clinical status (Parinni et al. Int. J. Mol. Sci. 2020;21(8):2975). [12] Recurrent respiratory infections, difficulty in standing and walking, and increased joint stiffness have been demonstrated in patients following cessation of ERT, thus emphasizing the need for continuous ERT. (Jurecka et al. Mol Genet Metab. 2012;107(3):508–12). [13]

Here we report successful long-term experience with the use of idursulfase beta in two adolescent Malaysian patients with MPS II, who experienced recurrent infusion-associated reactions warranting discontinuation of ERT with idursulfase.

## 2 Case presentations

### 2.1 Case 1

The first case is a follow-up of our previously published case report. (Ngu et al. 2017). [7] The patient is the elder of 2 boys of non-consanguineous parents, diagnosed with MPS II at the age of 6 years. The patient presented with frequent nasal congestion, snoring at night and progressive hepatomegaly at 21 months of age. A cardiac murmur due to mitral and aortic regurgitation was detected along with progressive joint contractures. Urinary GAG at diagnosis was 34.45 mg/mmol creatinine (reference range < 11 mg/mmol creatinine). Enzyme analysis performed on peripheral blood leukocytes showed undetectable iduronate 2-sulfatase activity (Normal range of iduronate 2-sulfatase assay National Referral Laboratory South Australia: 10.9–88.0 pmol/min/mg of protein). A hemizygous pathogenic mutation in exon 9 of the *IDS* gene (NM_000202: c.1608_1609delTA (p.Tyr536Ter)) was detected through molecular testing. (Ngu et al. 2017). [7]

At 11 years of age, the patient had mild obstructive sleep apnea and an enlarged liver palpable 5 cm below the right costal margin. Echocardiography revealed moderate aortic regurgitation with thickened aortic valve and mild mitral regurgitation. In September 2011, at 11 years of age, the patient was started on ERT with idursulfase (Elaprase) at 0.5 mg/kg (12 mg) weekly over 4 h with pre-medication of intravenous hydrocortisone, promethazine and oral loratadine. However, from the 26th infusion the patient developed extensive urticarial rash with idursulfase, not responding to treatment with antihistamines, despite reduction in dose and slower infusion rate. It was not possible to complete the 47th infusion due to recurrent urticarial rash and hypotension. The infusion at lower dose (1–6 mg/infusion) was resumed from the 48th–99th infusions, and it was identified that any dose >6 mg was associated with generalized urticaria through 100 infusions (Ngu et al. 2017). [7] Serum anti- idursulfase IgG antibodies, tested by conformation specific assay (enzyme-linked immunosorbent-based assay) and confirmed by radioimmunoprecipitation assay, were detected at the titer of 1:400, while IgE antibodies were not detected. Due to sub-optimal dosing and frequent interruption of ERT, there was minimal clinical improvement and urinary GAGs remained elevated at a range of 19.74–52.64 mg/mmol creatinine measured 12–24 months after infusion of idursulfase. (Ngu et al. 2017) [7]

**Fig. 1:**
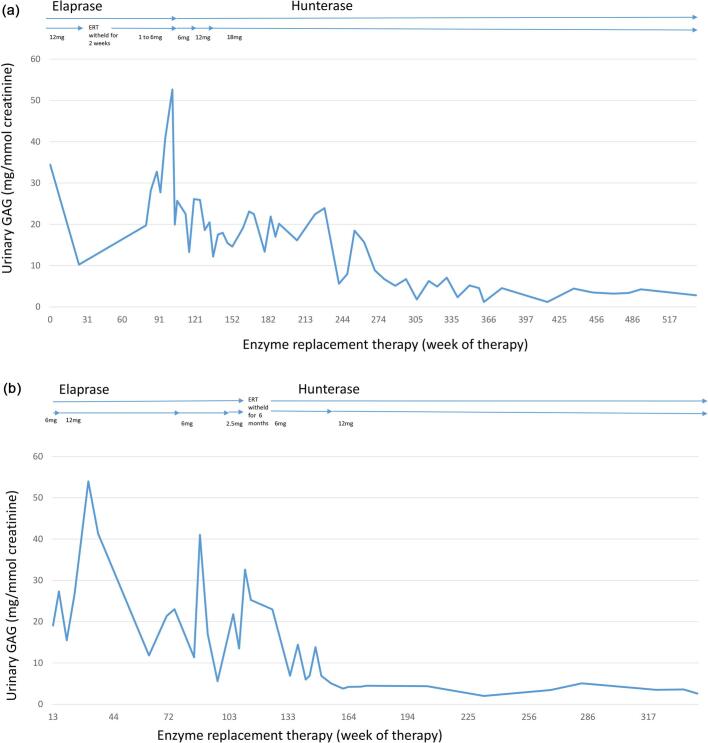
a: Urine GAG levels during ERT in patient 1 Urinary GAGs were high during suboptimal doses of IV idursulfase (Elaprase) when patient developed intolerable adverse drug reactions and urinary GAGs were low during optimal treatment dose with IV idursulfase beta (Hunterase) (12 and 18 mg = 0.5 mg/kg body weight). b. Urine GAG levels during ERT in patient 2. Urinary GAGs were high during suboptimal doses of IV idursulfase (Elaprase) when patient developed intolerable adverse drug reactions and urinary GAGs were low during optimal treatment dose with IV idursulfase beta (Hunterase) (12 mg = 0.5 mg/kg body weight).

At the age of 13 years (December 2013), the patient was switched to weekly idursulfase beta (Hunterase) infusions, and he received a gradually increasing dose from 6 mg/infusion to 18 mg/infusion (0.5 mg/kg/dose). To date (July 2022), the patient has been on idursulfase beta for 8 years 7 months and the treatment has been well tolerated with no serious infusion-related adverse reactions. The patient continues to remain stable with weight and height gain, stable cardiorespiratory status, and reduced urine GAGs of 2.01 mg/mmol creatinine. (Normal range < 11 mg/mmol creatinine). (Table 1, Fig. 1).

**Table 1:** Clinical assessment prior to ERT and after idursulfase (Elaprase) and idursulfase beta (Hunterase) treatment in patient 1.

	Baseline prior to commencement of ERT	After 24 months of idursulfase	After 24 months of idursulfase beta	After 8 years of idursulfase beta
	
Weight (kg)	26.3 (<3rd centile)	28 (<3rd centile)	38 (<3rd centile)	52 (<3rd centile)
Height (cm)	126 (<3rd centile)	133 (<3rd centile)	147 (<3rd centile)	162 (<3rd centile)
Head circumference (cm)	55 (98th centile)	54 (98th centile)	58 (>98th centile)	58.5 (98th centile)
Hepatomegaly (cm)	5	Not palpable	Not palpable	Not palpable
Splenomegaly (cm)	Not palpable	Not palpable	Not palpable	Not palpable
6MWT (m)	440	460	500	443
Echocardiography	LVEF 52%	LVEF 67%		LVEF 65%
	Mild MR with prolapsed AVML. Thickened aortic valve with moderate AR	Mild MR and AR	Mild MR and AR	Trivial MR, mild AR
	Dilated LA and LV	Dilated LA and LV	LA and LV not dilated	LA and LV not dilated
Sleep study	Mild obstructive sleep apnea	No obstructive sleep apnea	No obstructive sleep apnea	No obstructive sleep apnea
	O2 desaturation index 4.9	O2 desaturation index 0.5	O2 desaturation index 0.2	% predicted FVC 78%
				% predicted FEV1 67%
Neurological status	Cranial nerves intact	Cranial nerves intact	Cranial nerves intact	Cranial nerves intact
	Normal power, tone and reflexes	Normal power, tone and reflexes	Normal power, tone and reflexes	Normal power, tone and reflexes
	No sensory deficits	No sensory deficits	No sensory deficits	No sensory deficits
	No cognitive impairment	No cognitive impairment	No cognitive impairment	No cognitive impairment
		No change compared to baseline	No change compared to baseline	No change compared to baseline
Joint assessment				
Range of motion (Normal range)				
Shoulder flexion (180^o^)	135^o^	128^o^	120^o^	133^o^
Shoulder extension (45-60^o^)	30^o^	30^o^	30^o^	49^o^
Shoulder abduction (150^o^)	90^o^	115^o^	110^o^	110^o^
Elbow flexion (150^o^)	150^o^	120^o^	144^o^	145^o^
Elbow extension (−6-11^o^)	30^o^	35^o^	33^o^	35^o^
Hip flexion (120^o^)	100^o^	118^o^	95^o^	88^o^
Hip extension (16-20^o^)	5^o^	10^o^	10^o^	16^o^
Knee flexion (140-144^o^)	130^o^	112^o^	112^o^	111^o^
Knee extension (0^o^)	0^o^	0^o^	0^o^	0^o^
Urine GAG (mg/mmol creatinine) (Normal range < 11)	34.45	27.48	19.70	2.01
Anti-drug antibodies	Not determined	Anti-idursulfase antibodies	Not determined	Not determined
		IgG 1:400		
		Ig E not detected		

AR: aortic regurgitation; AVML: anterior mitral valve leaflet, FVC: forced vital capacity, FEV1: forced expiratory volume in one second; GAG: Glycosaminoglycans; LA: left atrium, LV: left ventricle; LVEF: left ventricular ejection fraction; MR: mitral regurgitation. 6MWT: 6-min walk test.

The patient had a WISC (Wechsler Intelligence Scale for Children) score of 97 (average IQ) at the age of 9 years prior to the start of ERT. MRI brain and spine performed at the age of 15 years showed mild vermis hypoplasia, and GAG deposition at the atlantoaxial joint without causing subluxation. No other abnormalities were noted. No significant abnormalities were noted in the MRI spine. He has average intelligence and has completed a diploma course. His current neurological examination is normal.

### 2.2 Case 2

Patient 2 is a 17-year-old boy with MPS II. He is the first child of non-consanguineous parents and has a younger brother who is similarly affected with MPS II. The patient was brought to medical attention at the age of 7 years when his mother noticed that he had short stubby fingers. Prior to that, he had recurrent upper respiratory tract infections, noisy breathing since infancy, umbilical hernia and bilateral inguinal hernia which was operated on at the age of 3 years. During the first medical consultation, the patient was noted to have short stature, macrocephaly, coarse facies, macroglossia, multiple joint contractures and large Mongolian blue spots over his back. Urinary GAGs were elevated, with raised dermatan sulfate and heparan sulfate. Enzyme analysis performed on peripheral blood leukocytes showed iduronate 2-sulfatase activity of 0.2 pmol/min/mg protein (Normal range 10.9–88.0 pmol/min/mg protein). Molecular analysis of the *IDS* gene identified a hemizygous mutation in the exon 3 of the *IDS* gene (NM_000202): c.329G > A (p.Arg110Lys).

Echocardiography revealed thickened mitral valve leaflets with anterior mitral valve prolapse and mild to moderate mitral regurgitation. Dysostosis multiplex was seen on skeletal survey. Ophthalmology examination showed no corneal clouding. MRI of the brain and cervical spine done at the age of 7 years showed bilateral enlarged lateral ventricles with prominent perivascular spaces. He had a hypoplastic dens and significant deposition of GAG around it which did not cause narrowing of the cervical cord. Pure tone and impedance audiometry revealed mild to moderate mixed hearing loss of the right ear, and mild to severe mixed hearing loss of the left ear. He was started on nocturnal continuous positive airway pressure (CPAP) at the age of 8 years for obstructive sleep apnea.

Treatment with idursulfase was initiated at the age of 9 years at the dose of 6 mg (0.25 mg/kg) weekly and was increased to 12 mg (0.5 mg/kg) weekly after 2 months. The patient was premedicated with oral prednisolone, cetirizine, promethazine and paracetamol 30 min prior to the infusion of idursulfase, which was given over 4 h. However, the patient developed urticarial rashes during the 81st infusion, which initially responded to a slower infusion rate and chlorpheniramine. The dose of idursulfase was lowered to 6 mg (0.25 mg/kg) from the 82nd to the 111th infusion. The urticarial rash persisted despite the reduced dose and reduced infusion rate. The 112th to 114th infusion could not be completed due to extensive urticarial rashes. The treatment was then withheld for the next 6 months. At the age of 12 years, the patient was recommenced on ERT but switched to idursulfase beta at a weekly dose of 6 mg (0.2 mg/kg) in January 2018. The dose was increased to 12 mg (0.5 mg/kg) from the 157th infusion till date. Infusions of idursulfase beta were well tolerated with no major infusion-related reactions. To date (July 2022), the patient has received 234 doses of idursulfase beta with only occasional mild transient urticaria. Urinary GAG levels have remained low throughout the treatment with idursulfase beta. The urinary GAG level reduced to 3.49 mg/mmol creatinine (Normal range < 11 mg/mmol creatine) after 36 months of idursulfase beta. (Table 2, Fig. 1).

**Table 2:** Clinical assessment prior to ERT and after idursulfase (Elaprase) and idursulfase beta (Hunterase) treatment in patient 2.

	Baseline prior to commencement of ERT	After 12 months of idursulfase	After 12 months of idursulfase beta	After 36 months of idursulfase beta
Weight (kg)	25.2 (25th centile)	28.6 (50th centile)	37.5 (25th centile)	39.4 (<3rd centile)
Height (cm)	118 (<3rd centile)	124 (<3rd centile)	131.5 (<3rd centile)	132 (<3rd centile)
Head circumference (cm)	58 (>98th centile)	58 (>98th centile)	58 (>98th centile)	58.5 (>98th centile)
Hepatomegaly (cm)	6	2	Not palpable	Not palpable
Splenomegaly (cm)	2	Not palpable	Not palpable	Not palpable
6MWT (m)	308	262	Not available	250.5
Echocardiography	LVMI 88.3 g/m^2^	Mild to moderate MR	MV myxomatous	Posterior MVL retracted, thickened leaflet
	LVEF 69.5%		LVEF 72%	Mild MR
	Mild to moderate MR		Mild MR, mild AR	Trivial AR, thickened leaflet
			Good biventricular function	Preserved biventricular function
Sleep study	Overnight pulse oximetry:	Overnight pulse oximetry normal	Desaturation index event 2.3	Not available
	Mean high 98.2%		Baseline SpO_2_95%	
	Mean low 92.5%		No major episodes of desaturation	
Neurological status	Cranial nerves intact	Cranial nerves intact	Cranial nerves intact	Cranial nerves intact
	Normal power, tone and reflexes	Normal power, tone and reflexes	Normal power, tone and reflexes	Normal power, tone and reflexes
	No sensory deficits	No sensory deficits	No sensory deficits	No sensory deficits
	No cognitive impairment	No cognitive impairment	No cognitive impairment	No cognitive impairment
		No change compared to baseline	No change compared to baseline	No change compared to baseline
Joint range of motion (Normal range)				
Shoulder flexion (180^o^)	80^o^	142^o^	132^o^	145^o^
Shoulder extension (45-60^o^)	40^o^	32^o^	40^o^	38^o^
Shoulder abduction (150^o^)	98^o^	130^o^	130^o^	140^o^
Elbow flexion (150^o^)	138^o^	130^o^	130^o^	120^o^
Elbow extension (−6-11^o^)	18^o^	28^o^	21^o^	25^o^
Hip flexion (120^o^)	115^o^	115^o^	105^o^	100^o^
Hip extension (16-20^o^)	15^o^	15^o^	15^o^	9^o^
Knee flexion (140-144^o^)	110^o^	118^o^	122^o^	118^o^
Knee extension (0^o^)	10^o^	10^o^	12^o^	11^o^
Urine GAG (mg/mmol creatinine) (Normal range < 11)	19.08	11.82	13.81	3.49
Anti-drug antibodies	Not determined	Not detected	Not determined	Not determined

AR: aortic regurgitation; GAG: Glycosaminoglycans; LVEF: left ventricular ejection fraction; LVMI: left ventricular mass index; MR: mitral regurgitation; MV: mitral valve; MVL: mitral valve leaflet; SpO2: pulse oximetry saturation. 6MWT: 6-min walk test.

He has a history of generalized tonic clonic seizures since the age of 17 years. MRI brain at 17 years of age showed communicating hydrocephalus with prominent subarachnoid spaces at bitemporal and posterior fossa regions. He has average intelligence and is currently attending mainstream secondary school. His current neurological examination is normal.

## 3 Discussion

Since MPS II is a life-limiting, multisystemic disease severely affecting the patients' quality of life; slowing or stopping the disease progression and improving quality of life are considered as successful clinical outcomes (Raimann et al. 2011), (Scarpa et al. Orphanet J. Rare Dis. 2011;6:72). [4,14] Development of successful ERT addressing the lysosomal enzyme deficiency has therefore been an important milestone in the management of MPS II. Clinical evidence demonstrates that ERT with idursulfase and idursulfase beta results in significant improvements in pulmonary function, endurance, joint mobility, and quality of life. (Muenzer et al. Genet Med. 2006;8(8):465–73) (Sohn et al. Orphanet J Rare Dis. 2013;8:42). [15] [16] Furthermore, reduction in spleen and liver size, reduction in urine GAGs, improvement in lung function and quality of life of both patients and family are sustained with continuation with ERT (Muenzer et al. Genet Med. 2011;13(2):95–101). [17]

Our case series presents long-term experience with idursulfase beta (Hunterase) in treating patients with severe MPS II. Patient 1 has been on ERT for 10 years 9 months from 2011 to 2022, out of which the patient received idursulfase for the first 2.2 years (116 weeks). To date, patient 1 has been on idursulfase beta for 8 years 7 months from December 2013 to July 2022. Idursulfase beta has been well-tolerated, and patient 1 continues to remain stable clinically with weight and height gain and reduced urine GAGs (Table 1) (Fig. 1). Patient 2 has been on ERT for over 7.5 years from January 2015 to July 2022, out of which the patient received idursulfase for the first 2.2 years (114 weeks), followed by a period of withdrawal before switching to Hunterase. To date, patient 2 has been on idursulfase beta for 4.5 years (234 weeks), which has been well-tolerated, and his joint motion, endurance, and other somatic symptoms remain stable. (Table 2) (Fig. 1).

For both patients, there was a significant improvement in the hepatosplenomegaly with idursulfase treatment, which was sustained during treatment with idursulfase beta. The cardiorespiratory function stabilized, and there was slight improvement and stabilization of the joint range of motion. However, patient 1 was noted to have reduction in hip flexion after long term treatment with idursulfase beta, which may account for deterioration in the 6-min walk test (6MWT). Patient 2 has limited bilateral ankle flexion, which may explain the reduction in distance of his 6MWT after 36 months treatment with idursulfase beta. Both patients' physiotherapy sessions were interrupted due to the movement restriction order imposed during the recent Covid-19 pandemic, which may have contributed to the worsening of their joint range of movement.

Despite reduction in dose, slowing of infusion rate, and premedication with corticosteroids and antihistamines, both our patients developed extensive urticarial rash with idursulfase, resulting in discontinuation of ERT. Treatment with idursulfase beta was well-tolerated by both patients with no serious infusion-related adverse reactions, allowing long-term continuation of ERT, with significant improvements in somatic symptoms, and quality of life. Our case series demonstrates the tolerability and safety of idursulfase beta in patients with MPS II after persistent intolerable adverse drug reactions with idursulfase and highlights that idursulfase beta offers another treatment option in MPS II. It could be considered as an alternative treatment in patients who cannot tolerate idursulfase or as a first line treatment in MPS II.

## Funding

This research did not receive any specific grant from funding agencies in the public, commercial, or not-for-profit sectors. GC Biopharma Corp. sponsored and supported the cost for preparing the manuscript.

## Declaration of Competing Interest

The authors declare that they have no conflict of interest.

## Data Availability

Data will be made available on request.
